# Radiological findings of malignant peripheral nerve sheath tumor: reports of six cases and review of literature

**DOI:** 10.1186/s12957-016-0899-0

**Published:** 2016-05-10

**Authors:** Yong-hui Yu, Jing-tao Wu, Jing Ye, Ming-xiang Chen

**Affiliations:** Department of Medical Imaging, Subei People’s Hospital of Jiangsu Province, 98# Western Nantong Road, Yangzhou, 225001 China

**Keywords:** Nervous-peripheral, Tomography, X-ray computer, MR imaging

## Abstract

**Background:**

Malignant peripheral nerve sheath tumor (MPNST) is a kind of rare neurogenic tumor. If associated with neurofibromatosis type 1, MPNST usually has a higher mortality. The aim of the article is to assess the imaging characteristics of MPNST and compare them with those of benign peripheral nerve sheath tumor (BPNST) to characterize this tumor.

**Methods:**

Clinical and imaging data of six cases with MPNST and 28 cases with BPNST in our institution since 2011 were retrospectively reviewed. Thirty-three patients have available MR imaging data, and two patients of MPNST also accepted CT scan. One patient accepted CT scan only. Location, size, shape, signal or density, boundary, bone destruction, relation to adjacent nerve, contrast-enhanced features as well as some other signs were assessed and compared with statistical software. Student’s *t* test was used for comparison of continuous variables. Fisher’s exact test was used for analysis of nominal variable. A *P* value ≤0.05 was considered to be statistically significant.

**Results:**

Differences existed between two groups in tumor size ((7.2 ± 3.3)cm in MPNST vs. (3.8 ± 1.4)cm in BPNST), unclear margin (4/6 in MPNST vs. 1/28 in BPNST), eccentricity to the nerve (1/6 in MPNST vs. 21/28 in BPNST), intratumoral lobulation (4/6 in MPNST vs. 2/28 in BPNST), peritumoral edema (3/6 in MPNST vs. 0 in BPNST), and peripheral enhancement (4/6 in MPNST (three of five MR, one CT) vs. 4/28 in BPNST). Bone destruction was observed in one MPNST.

**Conclusions:**

MR imaging is a valuable, non-invasive modality for the diagnosis of MPNST. Peripheral enhancement with non-cystic appearance or remarkable heterogeneous enhancement may be useful for differential diagnosis. Other imaging features such as large size (over 5 cm in diameter), ill-defined margin, intratumoral lobulation, peritumoral edema, and adjacent bone destruction are also supportive of MPNST.

## Background

Malignant peripheral nerve sheath tumor (MPNST) is a kind of highly aggressive neoplasm originated from Schwann cells. As the name implies, MPNST develops in the peripheral nerves and scarcely affects the cranial nerves [1].The total incidence is extremely low, about 0.001 % of the population [2].About 20–30 % of MPNST are seen in patient with neurofibromatosis type 1(NF-1) [3], with mortality up to 4.6–13 % [4]. Unresectable because of adjacency to big vessel or nerve trunk, frequent recurrence and distant metastasis of this tumor can be fatal. So, it is of great importance to diagnose MPNST before surgery, especially in NF-1 patients. Overlapping in clinical manifestations, distinguishing it from benign neurogenic tumor is difficult. Imaging modalities play an irreplaceable role in diagnosing, forming strategy of treatment as well as following up. X-ray examination has limitations except that mammography can be used for tumor in the breast [5]. Ultrasonography is an easy, cost-effective, and repeatable modality [6] but unsuitable for deeply located lesion, i.e., in the retroperinum and spine canal. PET/CT allows for sensitive whole-body scan for metastasis but lack of specificity. Computed tomography is effective in locating the tumor, giving an initial diagnosis and making plan for surgery. As a multi-parameter modality, MRI can provide abundant information on characterizing different components in tumor, thus is used for further examination. There are researches by many authors on imaging of MPNST; however, as its rarity and lack of specificity, imaging characteristics are not well known, and reports on superficial and retroperitoneal lesion are rare. In this article, we report imaging findings of six cases with histological proven MPNST, compare them with BPNST, and try to characterize this tumor.

## Methods

### Clinical data

The study was a retrospective analysis of patient data and requires no additional treatment, so ethical committee approval was waived. Written informed consents were obtained from all patients for publication of their clinical and imaging data. We reviewed 6 cases of MPNST and 28 cases of BPNST (including 26 cases of neurofibroma and 2 cases of schwannoma) in our institution since 2011. There were totally 15 males and 19 females, ranging in age from 23 to 78 years (52.8 ± 12.9 years in average). Clinical manifestations include enlarging palpable mass (9/34), pain or discomfort (9/34), cough (2/34), weakness (1/34), and frequent micturition (1/34). Twelve BPNSTs were discovered accidentally or through physical examination. Thirty-three patients have available MR imaging data, and two patients of MPNST also accepted CT scan. One patient accepted CT scan only. Two MPNST and two BPNST patients had neurofibromatosis type 1 at the same time. One MPNST in the upper arm had history of surgery in the same location (Table 1).

**Table 1:** Clinical data of MPNST and BPNST

Characteristic	MPNST (*n* = 6)	BPNST (*n* = 28)	*P*
Average age	60.7 ± 11.2	51.1 ± 12.8	0.101
Gender			0.328
Male	4	13	
Female	2	15	
Location			0.128
Paraspine	0	16	
Upper extremities	1	1	
Lower extremities	1	3	
Retroperinum	2	2	
Head and neck soft tissue	1	3	
Orbit	0	2	
Trunk	1	1	
NF-1	2	2	0.135
Symptom			0.059
Lump	4	5	
Pain or discomfort	1	8	
Physical examination	0	12	
Others	1	3	
Former surgery in same site	0	1	0.176

### Magnetic resonance imaging

MR scans were performed on two 3 T units (Signa HDx and Discovery MR750; General Electric Medical Systems, Milwaukee, WI, USA). MRI protocol included axial and coronal fat-saturated T2-weighted images, T1-weighted images, and post-contrast T1-weighted images. CT scans were performed with a 64-row scanner (LightSpeed VCT, General Electric Medical System). All examinations required contrast (gadopentetic acid salt, Schering, Germany or Iohexol, Yangtze, China) intravenous administration.

### Imaging and statistical analysis

Images were submitted to two experienced radiologists for review. The radiologists were required to obtain data on signal or density information. Signal or density characteristics were compared with those of muscle. Radiologists were also asked to assess location, size, shape, boundary, bone destruction, relations to adjacent nerve, contrast-enhanced features as well as some other signs (i.e. target sign, split fat sign). All data were obtained by consensus agreement.

Statistical analysis was performed with SPSS software (version 19.0). Student’s *t* test was used for comparison of continuous variables, which were expressed in form of mean and standard deviation values. Fisher’s exact test was used for analysis of nominal variable. A *P* value ≤0.05 was considered to be statistically significant.

## Results

### Locations

In MPNST group, there were six excised tumors in six patients, involving the knee, upper arm, cheek, waist, and retroperitoneal area, respectively. Most tumors developed in the subcutaneous tissue except for two retroperitoneal tumors, one of which was eccentric to the sciatic nerve. No split-fat sign is observed. As for BPNST group, there were 28 excised lesions in 28 cases, and mainly involves intermuscular space of the extremities, neck, and trunk, as well as the paraspinal, retroperitoneal region, and orbit. Connection between tumor and adjacent nerves can be seen in 21 cases (75 %), and split-fat sign was observed in 9 cases (32.1 %).

### Sizes

The average sizes of MPNST (7.2 ± 3.3 cm, range from 4.2 to 13 cm) were larger than that of BPNST (3.8 ± 1.4 cm, range from 1.1 to 6.3 cm).

### Shapes

A spindle or ovoid shape was noted in 22 BPNST (78.6 %) and five malignant tumors (83.3 %).The margin of the tumors tended to be ill-defined in four MPNST (66.7 %) and one BPNST (3.6 %). Intratumoral lobulation can be seen in four MPNST (80 %) and two BPNST (7.1 %).

### Signal intensity (density) and enhancement

**Fig. 1:**
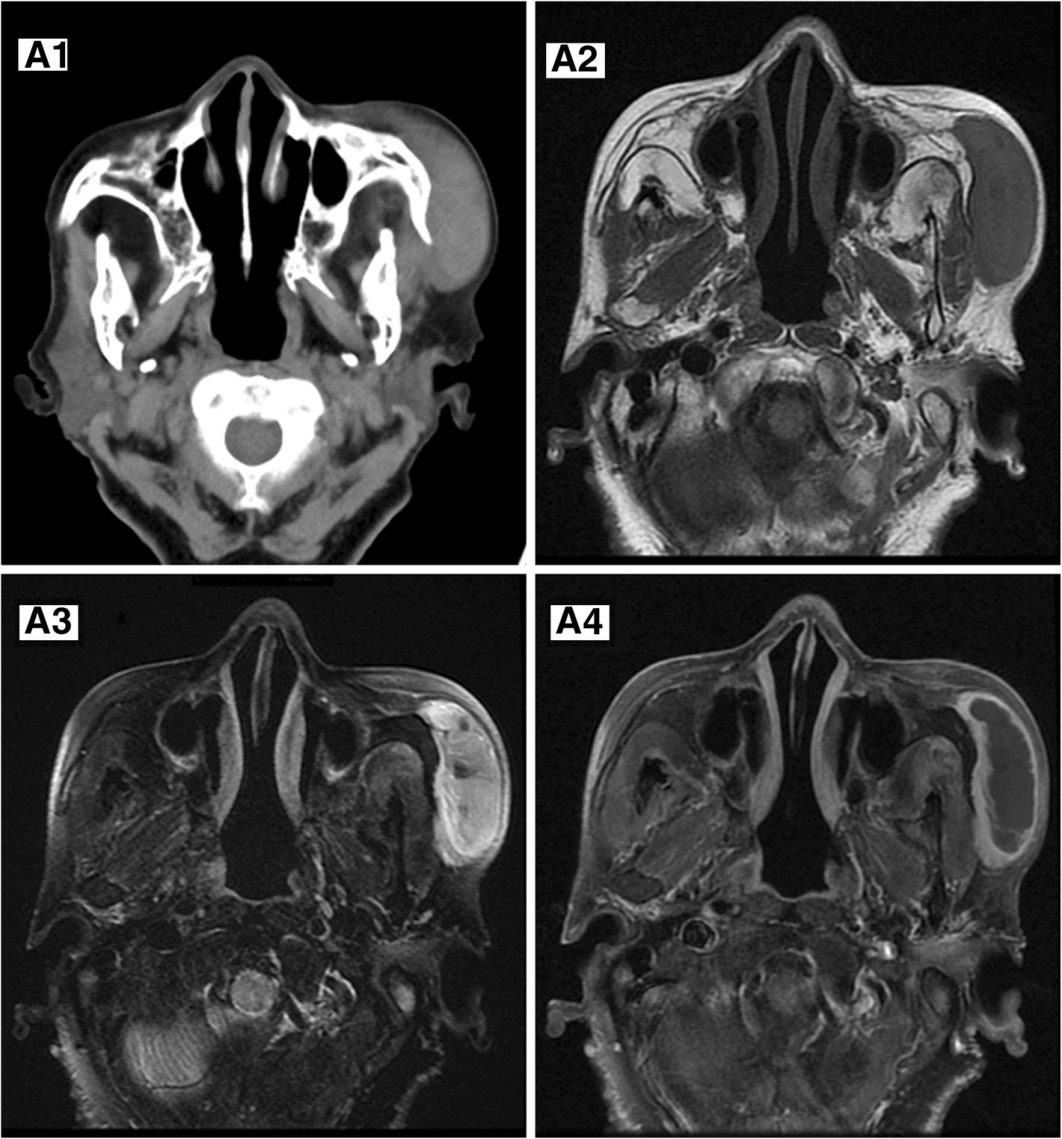
MPNST in a 65-year-old female. Plain CT scan (**A1**) shows a slightly hyper-attenuated lesion in the left cheek with slight zygomatic arch erosion. On MR scan, the tumor demonstrates hypo-intensity on axial T1-weighted (**A2**) and heterogeneous hyper-intensity on axial T2-weighted (**A3**) images. Gadolinium-enhanced T1-weighted images (**A4**) reveal peripheral enhancement. Note the central hyper-attenuated foci on plain CT and mixed-intense signal on T2WI, which is likely to be hemorrhage. Hemorrhagic cyst was conformed in surgery

**Fig. 2:**
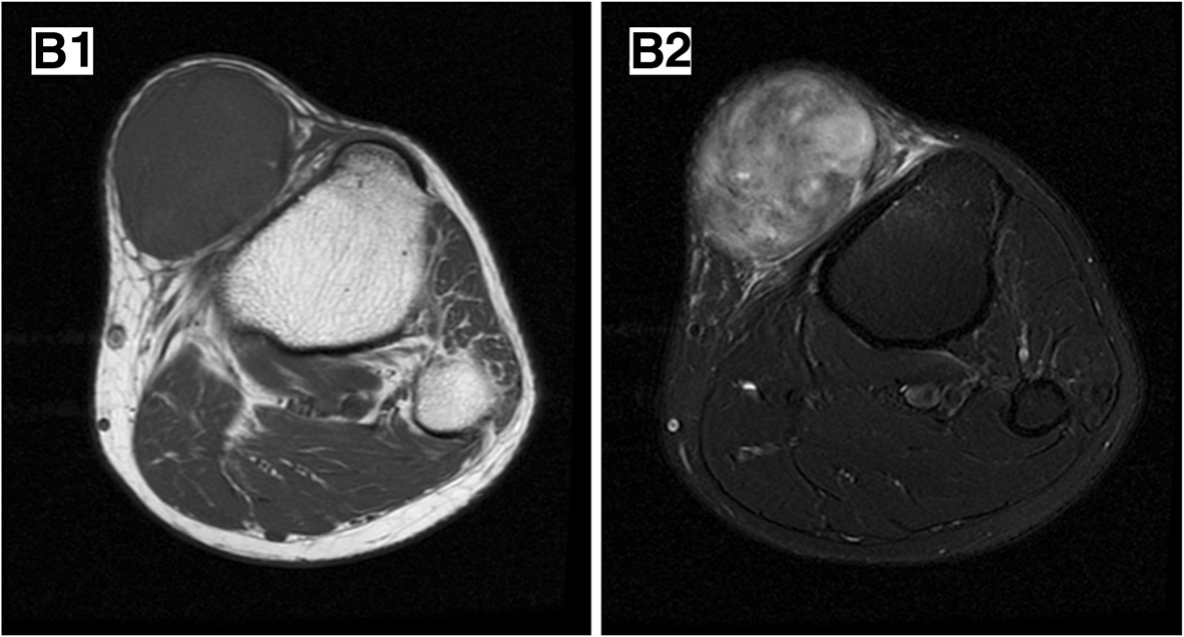
MPNST with NF-1 in a 49-year-old male. A well-defined, ovoid mass lies in the subcutaneous tissue, which demonstrates hypo-intensity on axial T1-weighted (**B1**) and obvious heterogeneous hyper-intensity on T2-weighted (**B2**) images. Perilesional edema and intratumoral lobulation are noticeable on T2WI

**Fig. 3:**
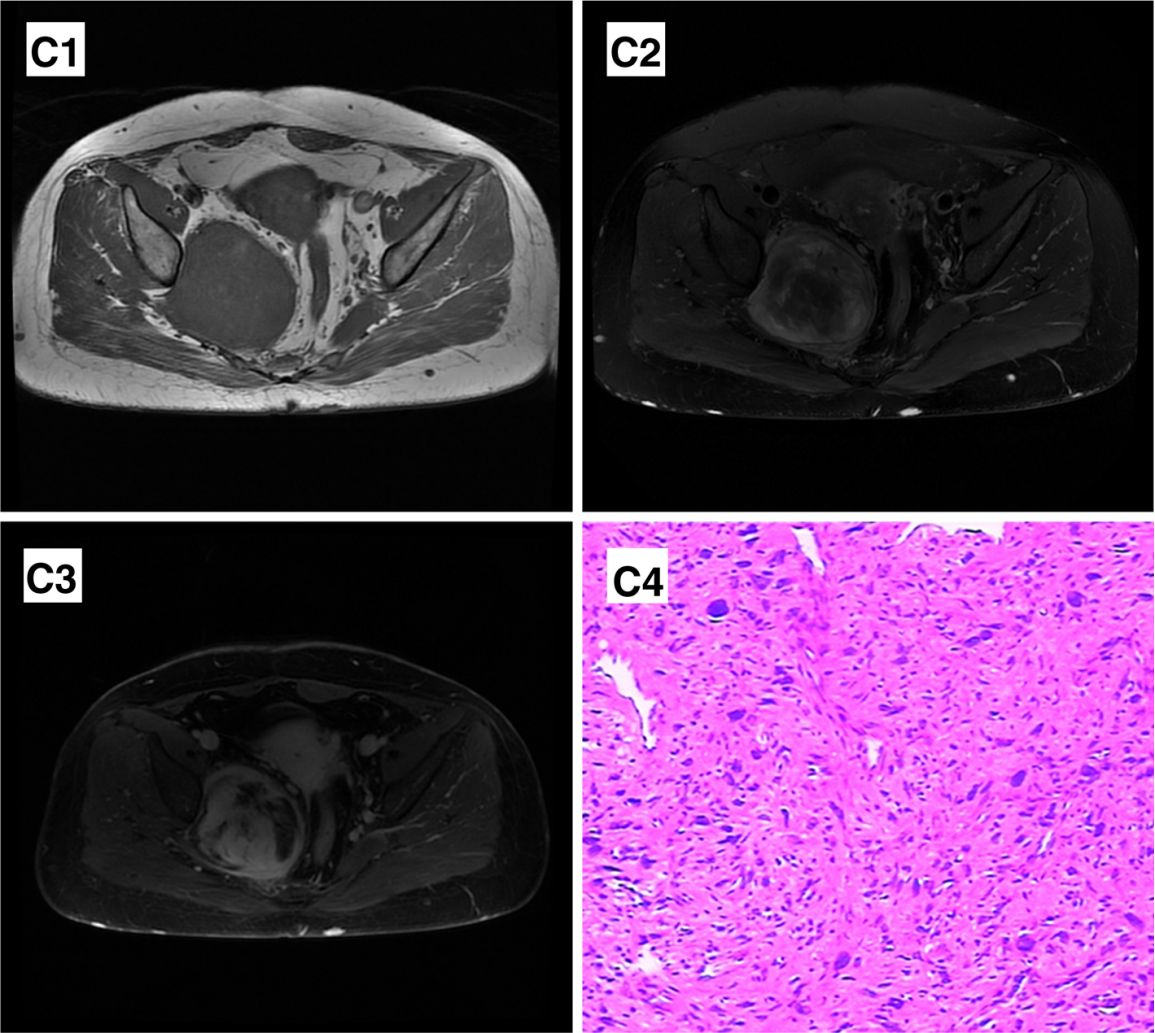
MPNST with NF-1 in a 47-year-old female. A well-defined, ovoid mass is located in the right pelvis wall, which demonstrates hypo-intensity on axial T1-weighted (**C1**) and obvious heterogeneous hyper-intensity on T2-weighted (**C2**) images. On enhanced T1 images (**C3**), there is strong enhancement in parts of the tumor with hypo-intense T2 signal, while moderate or slight enhancement in other parts. Note multiple subcutaneous neurofibromas. Hematoxylin-eosin staining result (100 times) (**C4**) shows prominence in nuclear mitosis activities and atypia

In MPNST group, four of five (80 %) tumors exhibited hypo-intense on T1-weighted images (T1WI) and hyper-intense signal on T2-weighted images (T2WI), and only one tumor demonstrated mixed signal on T1 and T2WI because of hemorrhage (Fig. 1 A2, A3). Two NF-1 associated tumors showed more complex signal on T2WI than other tumors (Figs. 2 B2, 3 C2). While in BPNST group, 20 (71.4 %) tumors were hypo-intense on T1WI and hyper-intense signal on T2WI, 2 (7.2 %) were mixed-intense signal, and 6 (21.4 %) were iso-intense on T1WI and hyper-intense signal on T2WI. Homogeneity of T1WI can be seen in 10 BPNSTs and 4 MPNSTs, whereas homogeneity of T2WI can only be seen in 1 BPNST. Typical target sign can be observed in five cases of BPNST but absent in all MPNST cases. Adjacent soft tissue swelled and exhibited hyper-intense signal on T2WI only in three cases of MPNST (Fig. 2 B2). On plain CT scan, density of tumors tended to be similar to that of the muscle. In a facial MPNST, calcification, hemorrhage, and adjacent bone erosion can be found (Fig. 1 A1). Enhancing pattern on CT and MR was much alike. After contrast administration, MPNST showed peripheral enhancement (Figs. 1 A4, 4 D2) (three of five MR, one CT) or irregular enhancement (Fig. 3 C3) (two of five MR). Peripheral enhancement was seen more often in MPNST than in BPNST (4 of 28).

**Fig. 4:**
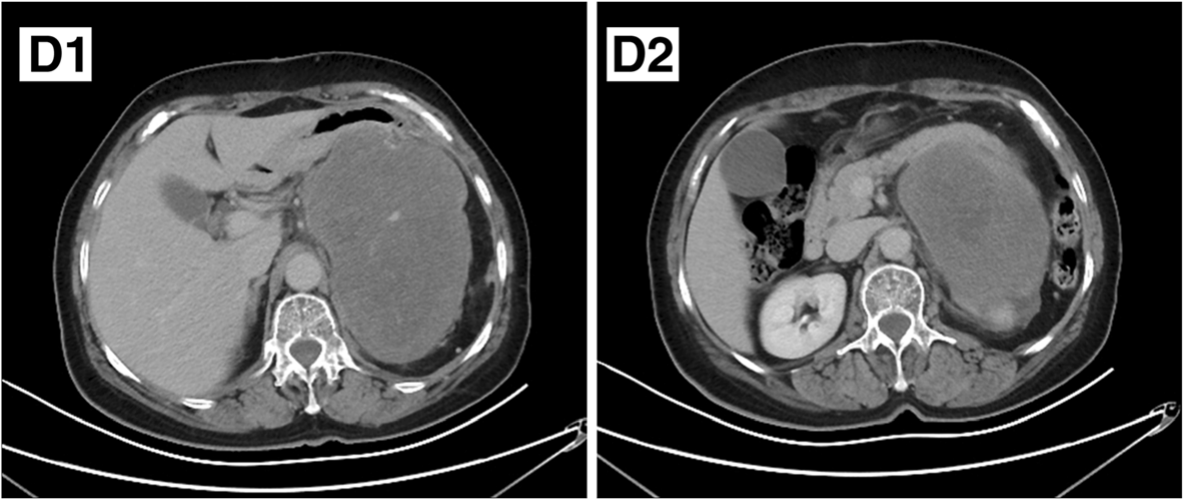
(**D1**) A huge, well-margined retroperitoneal MPNST is shown on enhanced CT scan, with feeding vessels inside. (**D2**) On the lower level, a hypo-attenuated foci can be observed, which was turned out to be a myxoid cyst

Differences between two groups were statistically significant in tumor size, margin, eccentricity to the nerve, peritumoral edema, intratumoral lobulation, and peripheral enhancement. No obvious difference existed between two groups in T1 and T2 signal intense, target sign, split-fat sign, and cystic change (Table 2).

**Table 2:** Comparison of imaging manifestations between BPNST and MPNST

	BPNST (*n* = 28)	MPNST (*n* = 6)	*P* value
Mean ± SD average size (cm)	3.8 ± 1.4	7.2 ± 3.3	0.002^a^
Shape			0.640
Oval or spindle	22 (78.6 %)	5 (83.3 %)	
Irregular	6 (21.4 %)	1 (16.7 %)	
Margin			0.002^a^
Ill-defined	1 (3.6 %)	4 (66.7 %)	
Well-defined	27 (96.4 %)	2 (33.3 %)	
Eccentricity to the nerve	21 (75.0 %)	1 (16.7 %)	0.014^a^
Sign on MRI (MPNST (*n* = 5))			
Hypo-intense T1 and hyper-intense T2	20 (71.4 %)	4 (80 %)	0.052
Mixed-intense T1 and T2	2 (7.2 %)	1 (20 %)	0.400
Iso-intense T1 and hyper-intense T2	6 (21.4 %)	0 (0 %)	0.340
T1 (homogenous)	10 (53.6 %)	4 (80 %)	0.089
T2 (homogenous)	1 (3.6 %)	0 (0 %)	0.848
Target sign	5 (17.9 %)	0 (0 %)	0.414
Split-fat sign	9 (32.1 %)	0 (0 %)	0.179
Intratumoral lobulation	2 (7.1 %)	4 (66.7 %)	0.004^a^
Cystic change	14 (50 %)	2 (33.3 %)	0.389
Perilesional edema-like zone	0 (0 %)	3 (50 %)	0.003^a^
Enhancement			
Peripheral with cystic change	4 (14.3 %)	4 (66.7 %)	0.018^a^
Honeycomb-like	9 (32.1 %)	0 (0 %)	0.132
Central	5 (17.9 %)	0 (0 %)	0.353
Homogeneous	3 (10.7 %)	0 (0 %)	0.547
Irregular	7 (25.0 %)	2 (33.3 %)	0.513

^a^Statistically significant

### Surgical and pathological findings

Four superficial tumors and one retroperitoneal tumor in MPNST group had grayish or yellowish appearance. One facial mass became partially cystic and dark red because of hemorrhage. Under microscope, they were all composed of intersecting fascicles of spindle cells alternated with hypocellular areas. In two NF-1 related tumors, there was more prominent mitosis and atypia in nuclear as well as necrosis (Fig. 3 C4). Immunohistochemical examinations were found positive for vimentin and S-100 protein. The retroperitoneal tumor in the upper abdomen was a lobulated mass surrounded with capsule. Flesh, myxoid cystic content was found in gross inspection. Adjacent spleen and pancreas tail were involved. Pathological findings revealed MPNST with glandular differentiation, immuno-histochemical results showed positive reaction of CK, NSE, and CD68.

## Discussion

### Clinical data

MPNST is a kind of rare, aggressive neurogenic tumor, accounting for only 5–10 % of soft tissue sarcomas [7]. It usually originates from the peripheral nerves, and in rare occasions, from malignant transformation of benign neurogenic tumor. The most common anatomic sites include proximal portion of the upper extremities, lower extremities and trunk. Redzepagic et al. [5] reported a case in the breast, which is a rare location for MPNST. Rafailidis et al. [6] believe that MPNST typically affects major nerve trunks like the sciatic nerve, the brachial plexus, and the sacral plexus. However, in our group, except for two retroperitoneal tumors, all MPNSTs developed superficially and had no direct connection with these major nerve trunks. MPNST usually affects patients in 20 to 50 years old, without gender predilection [6]. It occurs more often in association with NF-1 and leads to poor prognosis. Two MPNSTs in our group with history of NF-1 were more aggressive, with histologically more nuclear mitosis activities and obvious atypia.

### Radiological features

#### Size

Probably due to rapid growth, average size of MPNST is usually above 5 cm and larger than that of BPNST [8].

#### Boundary

MPNST can infiltrate into the surrounding soft tissue and cause peritumoral edema, which makes the boundary unclear (Fig. 2). Benign neurogenic tumor tends to be well defined and usually surrounded with capsule. However, some plexiform neurofibromas also have infiltrative appearance. When grows deeply, MPNST may be well margined. Two retroperitoneal MPNSTs in our study were well defined because they were surrounded with fat tissue, and capsule was founded in one abdominal lesion in surgery, possibly formed by compression of surrounding tissue in rapid growth. Ill-defined margin and peritumoral edema can be a useful sign but lack of specificity.

#### Relation with adjacent tissue

Spilt-fat sign is often seen in benign neurogenic tumor. MPNSTs in our group grew in subcutaneous tissue or retroperitoneal region, so this sign is irrelevant. Most of BPNST in our group were eccentric to adjacent nerves. Although developed in the peripheral nerve, contiguity with adjacent nerves was not observed in most MPNST cases. Perhaps they were originated from the minor branches of the nerve, which cannot be observed clearly in imaging and macro-examination. Li et al. [9] believed that contiguity with a specific nerve may support the diagnosis of a BPNST rather than a malignant one. This result is partly in agreement with our study, but we believe this sign is largely dependent on the location of the tumor.

#### Signal or density

On T1WI images, 18 BPNSTs showed inhomogeneous signal because of enlarged vessels. Signals of MPNST were relatively homogenous, only one tumor showed mixed-intense signal (focal high signal) because of hemorrhage. Matsumine et al. [10] thought that the presence of high signal on T1WI is a diagnostic indicator. Hemorrhage is also seen in schwannoma, so this sign may be not so specific. On T2WI images, in BPNST group, signals of 20 tumors were hyper-intense, and 2 tumors were mixed-intense because of enlarged vessels and deposition of hemosiderin after hemorrhage. There were different types of signal, including target sign, single or multiple cystic appearance, and homogenous signal. MPNST usually shows inhomogeneous signal, especially in the context of NF-1. This sign is in accordance with its malignant nature and indicates a poor prognosis. Two NF-1-associated MPNSTs in our study had complex signal on T2WI and were histologically more aggressive. Because there is only short-term follow-up since surgery, the outcomes are still unknown.

Target sign (peripheral high signal and central low signal on T2WI) is a characteristic sign of neurogenic tumor [9]. It is attributed to central fibrocollagenous tissue and peripheral myxomatous tissue [10]. It is rarely seen in MPNST. In our group, no typical target sign can be found in MPNST. But in BPNST group, only five cases showed this sign. So, we believe that target sign plays a limited role in differential diagnosis. Demehri et al. [11] studied 9 MPNSTs and 22 BPNSTs and found no significant difference in the presence of a target sign between them.

Intratumoral lobulation can also be detected in MPNST. It is considered to originate from a network-like growth of plexiform neurofibromas involving multiple fascicles and/or branches of a nerve, leading to a diffuse mass of thickened nerves [12]. In our group, there were four cases of MPNST and two cases of BPNST showing this sign (Fig. 2).

#### Enhancement

In contrast to central enhancement of BPNST, MPNST often shows peripheral enhancement on contrast-enhanced T1-weighted images [13]. In five BPNSTs with target sign of our group, there was strong enhancement in the center and slight enhancement in the peripheral region, that is to say, opposite signal to T2WI. It can be explained by more blood supply needed for tumor cells in the centrally densed area. In MPNST group, there was no such sign. Four MPNSTs enhanced significantly with foci lack of perfusion, which can be explained by cystic change or necrosis (Fig. 1). There was a case of MPNST with glandular differentiation in our group (Fig. 4), which showed consistent enhancement with tumor vessels inside. In surgery, cystic content was found. After a careful review of the images, we can find a hypo-attenuated area in tumor. Schwannoma can demonstrate peripheral enhancement because of cystic change, but rarely in neurofibroma. Wasa et al. [14] believed that intratumoral cystic change can assist in the differentiation of neurofibroma from MPNST. In our point of view, there may be a cyst formation in MPNST with peripheral enhancement, but the signal is complex on T2WI because of hemorrhage. This is different from hyper-intense signal in cystic formation. Peripheral enhancement with non-cystic appearance may be a valuable sign of MPNST. There was remarkable heterogeneous enhancement in two NF-related MPNST cases (Fig. 3), which can also be a sign of malignancy.

#### Brief introduction of previous studies

As for signs that are criminative between MPNST and BPNST, there are different points of view (Table 3). Chhabra et al. [4] laid a great importance on distinguishing MPNST between NF-1 and non-NF-1 patient and stressed that ill-defined margins and/or invasion of adjacent structures are highly specific for malignancy. Li et al. [9] reviewed 16 schwannomas, 1 neurofibroma, and 9 MPNSTs and draw a conclusion that larger size and infiltrative margin can be suggestive of malignancy. Matsumine et al. [10] analyzed data of neurofibroma and MPNST in 37 NF-1 patients and concluded that intratumoral lobulation and presence of high signal on T1WI were indicators of MPNST. Demehri et al. [11] studied 31 peripheral nerve sheath tumors, and hence, reached a conclusion that average tumor diameter and minimum ADC values are potentially important parameters. Derlin et al. [13] studied 67 BPNSTs, 8 MPNSTs, and decided that intratumoral lobulation, ill-defined margins, and irregular enhancement on T1WI were significantly associated with MPNSTs. Matsumoto et al.’s work [15] elaborated on dumbbell schwannomas and MPNSTs and stressed the importance of large maximal diameter, irregularly lobulated shape, boundary indistinguishable from surrounding tissues, and osteolytic bone destruction. Their studies are largely the same, focusing on tumor size, boundary, and intratumoral lobulation, which are lack of specificity. In a detailed study, Wasa et al. [14] believed that two or more of four signs (largest dimension, peripheral enhancement, perilesional edema, and cystic lesion.) may support the diagnosis of MPNST and stressed the meaning of peripheral enhancement. In our study, we made further investigation and combined peripheral enhancement with characteristic of signal.

**Table 3:** Imaging findings supportive of MPNST based on studies of different authors

	Matsumoto et al.	Chao-siang Li et al.	Matsumine et al.	Wasa et al.	Derlin et al.	Demehri et al.	Chhabra et al.
BPNST	15	17	18	20	67	22	35
MPNST	8	9	19	41	8	9	21
Irregular shape	√		√				
Large size	√	√		√		√	
Unclear margin	√	√	√		√		√
Cystic change				√			
Intratumoral lobulation		√	√		√		
No target sign			√				
Peritumoral edema				√			
Irregular enhancement			√				
Peripheral enhancement				√			

## Conclusions

MR imaging, as a noninvasive imaging technique, plays an important role in diagnosis before surgery. MPNST has diversity in imaging manifestations. Peripheral enhancement with non-cystic appearance and obvious heterogeneous enhancement may be valuable in diagnosis. Besides that, we believe, several points could be helpful to differentiate MPNST from BPNST: (1) over 5 cm in size and ill-defined, (2) peritumoral edema, when located superficially, (3) intratumoral lobulation, (4) absence of target sign, and (5) bone destruction is an indicative for malignancy. Except for the last sign, no single sign is enough for diagnosis. A combination of two or more of these features can facilitate us in early diagnosis and improve the prognosis, especially in patient with NF-1.

This study had some limitations. As MPNST is rare, only six cases were inrolled in the study, making the study less persuasive. But after careful comparisons with benign tumors and detailed review of literature, there is still some meaning for diagnosis.

## Ethics approval and consent for publication

Because this study involves no experiment, ethics approval is waived. Written informed consents were obtained from all patients for publication of their clinical and imaging data.

## Abbreviations

BPNST: benign peripheral nerve sheath tumor
CK: cytokeratin
MPNST: malignant peripheral nerve sheath tumor
MRI: magnetic resonance imaging
NF-1: neurofibromatosis type 1
NSE: neuron-specific enolase
T1WI: T1-weighted image
T2WI: T2-weighted image
